# Diagnostic Utility of High Sensitivity Troponins for Echocardiographic Markers of Structural Heart Disease

**DOI:** 10.3390/medsci6010017

**Published:** 2018-02-15

**Authors:** Tom Kai Ming Wang, Clementina Dugo, Gillian Whalley, Yvonne Wynne, Heather Semple, Kevin Smith, Peter Cleave, Jonathan Christiansen, Andrew To, Nezar Amir, Tony Scott, Ross Boswell, Patrick Gladding

**Affiliations:** 1 Lakeview Cardiology Centre, North Shore Hospital, Auckland 0620, New Zealand; Yvonne.Wynne@waitematadhb.govt.nz (Y.W.); Heather.Semple@waitematadhb.govt.nz (H.S.); Kevin.Smith@waitematadhb.govt.nz (K.S.); Jonathan.Christiansen@waitematadhb.govt.nz (J.C.); Andrew.To@waitematadhb.govt.nz (A.T.); Nezar.Amir@waitematadhb.govt.nz (N.A.); Tony.Scott@waitematadhb.govt.nz (T.S.); Patrick.Gladding@waitematadhb.govt.nz (P.G.); 2 Green Lane Cardiovascular Service, Auckland City Hospital, Auckland 1023, New Zealand; 3 Division of Cardiology, Azienda Ospedaliera Universitaria Integrata, 37126 Verona, Italy; Clementina.Dugo@waitematadhb.govt.nz; 4 Unitech University of Technology, Auckland 1025, New Zealand; G.Whalley@auckland.ac.nz; 5 Department of Pathology, Middlemore Hospital, Auckland 2025, New Zealand; Peter.Cleave@middlemore.co.nz (P.C.); Ross.Boswell@middlemore.co.nz (R.B.)

**Keywords:** troponin, echocardiography, structural heart disease, left ventricular hypertrophy

## Abstract

The conventional use of high-sensitivity troponins (hs-troponins) is for diagnosing myocardial infarction however they also have a role in chronic disease management. This pilot study assessed the relationship of hs-troponins with echocardiographic markers of left ventricular hypertrophy (LVH) and structural heart disease (SHD). Patients undergoing computer gomography (CT) coronary angiogram for low-intermediate risk chest pain and healthy volunteers were recruited. Hs-troponins Singulex I, Abbott I and Roche T and N-terminal pro-brain natriuretic peptide (NT-proBNP) were evaluated in relation to SHD parameters including left ventricular hypertrophy (LVH_Echo_) and left atrial enlargement (LAE_Echo_) on echocardiography. 78 subjects who underwent echocardiography were included in this study. C-statistics (95% confidence interval) of the four biomarkers for predicting LVH_Echo_ were 0.84 (0.72–0.92), 0.84 (0.73–0.92), 0.75 (0.63–0.85) and 0.62 (0.49–0.74); for LAE_Echo_ 0.74 (0.6–0.85), 0.78 (0.66–0.88), 0.55 (0.42–0.67) and 0.68 (0.62–0.85); and composite SHD 0.79 (0.66–0.88), 0.87 (0.75–0.94), 0.62 (0.49–0.73) and 0.74 (0.62–0.84) respectively. Optimal cut points for SHD were >1.2 ng/L, >1.6 ng/L, >8 ng/L and >18 pmol/L respectively. These results advocate the potential role of hs-troponins as screening tools for structural heart disease with theranostic implications.

## 1. Introduction

Troponins assays have revolutionised the diagnosis of myocardial necrosis and infarction [1]. However new uses for high sensitivity troponin are emerging, which may have a greater impact in primary care. These new uses have come about partly due to the fact that high-sensitivity troponin (hs-troponin) assays are able to measure troponin precisely, at low concentrations, even within a seemingly normal healthy population [2,3]. More importantly however is that troponin has a low index of individuality, meaning that variability within an individual over time is relatively narrow and therefore highly personalized [4,5,6]. Elevated troponin I has been associated with various disease states such as aging [3], hypertension [7], left ventricular (LV) hypertrophy [8], LV systolic dysfunction [9,10] and stable coronary artery disease [11].

Troponins have both diagnostic and prognostic capacity and are independently associated with the development of hypertension, heart failure, cardiovascular events, stroke and total mortality [12,13]. With respect to hypertension a recent study has shown that troponin T is elevated with both increasing diastolic blood pressure (DBP) and DBP <60 mmHg [14], with those elevations being associated with increased cardiovascular events, consistent with the J-shaped curve hypertension hypothesis. Similarly, increased troponin has been shown to be not only predictive for the presence of coronary artery disease but also myocardial infarction and death in those with established coronary artery disease [2]. Furthermore, treatment with a statin was associated with a reduction in troponin concentration, which corresponded with improved outcomes, independent of low density lipoproetin lowering in both the WOSCOPs and JUPITER studies [15,16].

The results of these studies demonstrate an emerging role of troponin as a longitudinal biomarker of cardiovascular risk and treatment response. However, many of these population studies and lack coronary or LV imaging information on patients. There are few studies where multimodal imaging has been applied alongside high sensitivity troponin testing. Whilst it is accepted that not all troponin assays are equivalent there have also been few studies comparing the utility of multiple contemporary troponin assays in parallel with multimodal imaging. We therefore undertook a pilot study employing two contemporary troponin assays and a research assay, using single molecule detection, in a group of patients undergoing computed tomography (CT) coronary angiography, echocardiography and multi-omic analysis.

## 2. Materials and Methods 

### 2.1. Study Population

The myHealth e-Body study comprised of 200 patients undergoing CT coronary angiogram for low-intermediate risk chest pain. This interim analysis focuses on troponins and imaging markers of structural heart disease and reports on 78 patients from this study, including healthy volunteers who did not undergo CT. Institutional ethics approval was attained prior to the commencement of the study (Health and Disability Ethics approval #15/NTB/34/AM03). All subjects gave written informed consent. Clinical characteristics collected included demographics, past medical history, 5-min electrocardiogram, a coronary artery and atrial fibrillation genetic risk score, metabolomics, echocardiography, CT coronary angiography and troponin assays and long-term outcomes including atrial fibrillation, cardiovascular events and mortality. Healthy volunteers did not undergo CT coronary angiography.

### 2.2. Echocardiography

Echocardiography was performed by a single Level III trained cardiologist, using a Philips CX50 machine (Philips, Amsterdam, Netherlands). Echocardiographic markers of structural heart disease examined included left ventricular hypertrophy (LVH_Echo_) defined as an interventricular septal thickness ≥11 mm and left atrial enlargement (LAE_Echo_) defined as ≥34 mls/m^2^, indexed to body surface area (BSA). Left ventricular global longitudinal strain and an echocardiographic calcium score was also applied but is described in another paper. 

### 2.3. Computed tomography Coronary Angiography 

Computed tomography Coronary Angiography (CTCA) was acquired using a 320-slice Aquillon ONE CT scanner (Toshiba Medical Systems, Otawara, Japan). Oral and intravenous Metoprolol were used according to institutional protocol to achieve a scanning heart rate of <65 beats per minute. Obstructive coronary artery disease was defined as the presence of coronary stenosis of diameter loss >50%. Vitrea automated software was used for LV mass calculation, with LVH_CT_ hereby defined as LV mass >81.7 g/m^2^, based on a study indicating adverse outcomes in patients with an LV mass above this cut point [17]. 

### 2.4. Troponin and NT-BNP Assays

Blood was collected and after centrifugation at 3000 *g*, for 5-min plasma was stored at −80 degrees centigrade before being shipped on dry ice to core lab facilities for testing. The fourth generation hs-troponin assays used included Singulex Erenna SMC troponin I (Limit of detection 0.04 ng/L, inter-/intraassay coefficient of variation 6% at 8.3 ng/L), Abbott Architect_STAT_ troponin I (Limit of detection 1.2 ng/L, interassay coefficient of variation <10% at 4.7 ng/L) and Roche Elecsys troponin T (Limit of detection 5 ng/L, interassay coefficient of variation 10% at 13 ng/L). N-terminal pro-brain natriuretic peptide (NT-proBNP) was measured using a Roche Elecsys assay on a Cobas 6000 analyser (Measuring Range 5–35,000 ng/L, intermediate precision 2.9–6.1%). Singulex troponin concentrations <0.19 ng/L, Abbott troponin I < 0.2 ng/L and Roche troponin T <0.3 ng/L were reported as absolute values corresponding to the lower cut-off for detection.

### 2.5. Statistics

Median (lower quartile, upper quartile and frequency (percentage) were used to present continuous and categorical variables respectively. Univariate analysis was performed using student *t*-test for continuous variables and Spearman coefficient (95% confidence intervals (CI)) for correlations. Receiver-operative characteristics curve (ROC) analysis was used to assess performance of troponin assays at predicting echocardiographic markers by c-statistic and identifying the optimal cut point (maximal sensitivity × specificity). A Bland Altman plot was used to demonstrate differences between the Singulex and Abbott troponin assays. Medcalc software version 16.8.4 (MedCalc Software, Ostend, Belgium) was used to analyse the data. All tests were two-tailed and *p* < 0.05 deemed statistically significant. 

## 4. Results

### 4.1. Cohort Characteristics

Diagnostic quality echocardiographic data was available for 78 patients. Cardiac CT was available for 62 (79%) patients, though LV mass could not be calculated for two patients due to poor contrast opacification of the LV. Eleven (14%) subjects were healthy volunteers and underwent an echocardiogram but not a CTCA. Measures of interventricular septal diameter (IVSd) were available in 75 (96%) of patients whereas echocardiographic LV mass could only be calculated in 64 (82%). Baseline characteristics for all patients are shown in Table 1. Amongst the clinical non-imaging characteristics, age (Spearman coefficient 0.27 (0.02–0.49), *p* = 0.03), male (3.63 vs. 1.21 ng/L, *p* = 0.008) and hypertension (2.95 vs. 0.97 ng/L, *p* = 0.006) were significantly associated with higher high-sensitivity troponins (Singulex assay). 

**Figure 1:**
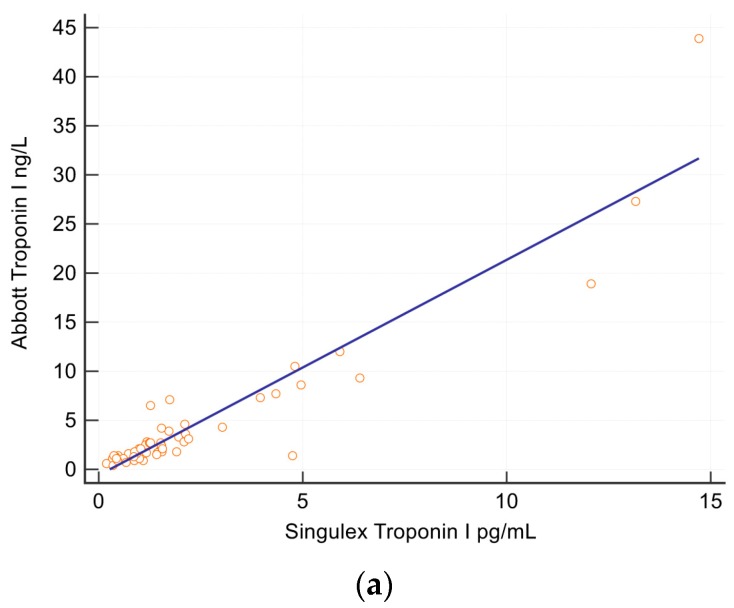

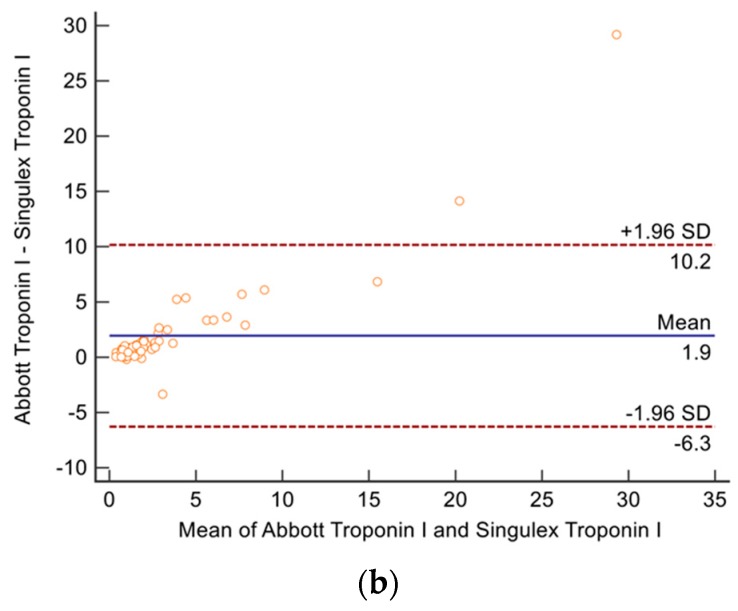
Correlation between Singulex Troponin I versus Abbot Troponin I. (**a**) Regression between Singulex Troponin I versus Abbott Troponin I. Spearman coefficient R^2^ = 0.87, *y* = −0.6106 + 2.1960*x*, *p* < 0.0001. (**b**) Bland Altman plot comparing Singulex and Abbott Troponins.

Cardiac characteristics of the whole patient cohort are also found in Table 1. Median hs-troponin levels were 1.2, 1.8 and 6.8 ng/L for Singulex, Abbott and Roche assays. Median (quartile 1 and 3) interventricular septal dimension was 0.9 (0.8–1.1) cm and left atrial volume index 32 (26–37) cm^2^/kg/m^2^. Correlation was strong between the two hs-troponin I assays, Spearman coefficient R^2^ = 0.87 (Figure 1) but weaker between I and T assays R^2^ = 0.37/0.54 (Abbott/Singulex) and weakest between hs-troponin and NT-proBNP assays R^2^ = 0.3/0.31. 

### 4.2. Predictors of Structural Heart Disease

LV systolic dysfunction (LVSD) was present in 8 (11%) patients, mean ± standard deviation (SD) ejection fraction 34 ± 6% versus 56 ± 3%, *p* < 0.0001. NT-proBNP had a high sensitivity and specificity for the detection of moderate-severe LV systolic dysfunction as shown in Table 2.

**Figure 2:**
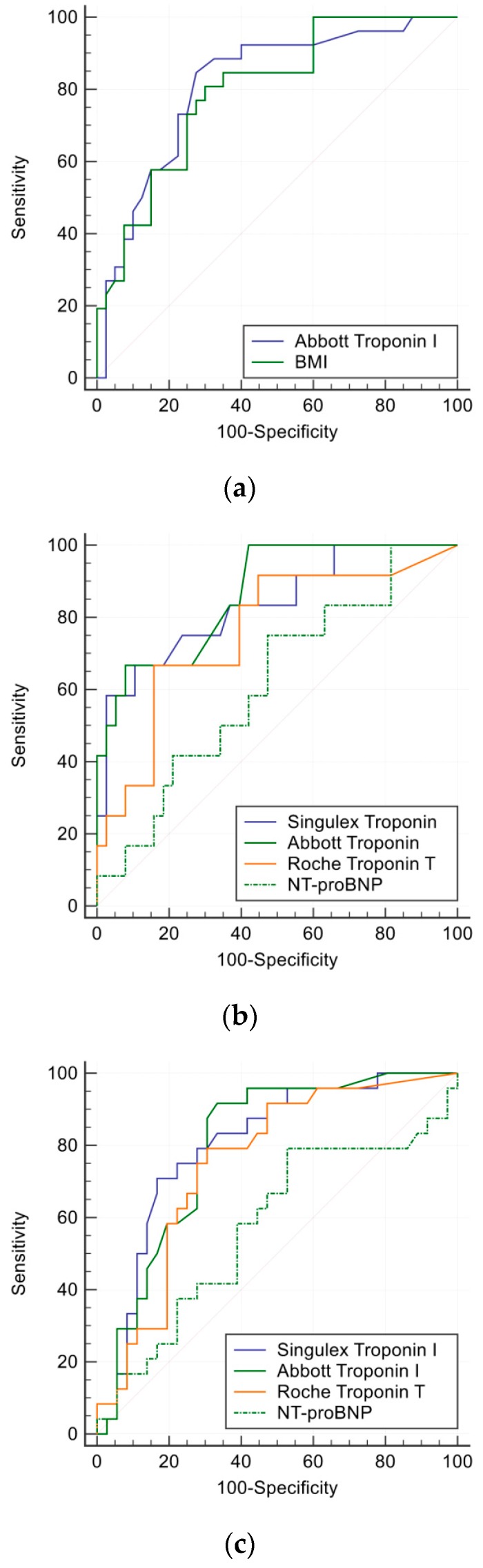
Receiver-operative characteristics analyses curves: (**a**) comparing Abbott troponin I and body mass index in prediction of LVH_Echo_, (**b**) biomarkers predictive of LVH_Echo_, (**c**) biomarkers predictive of LVH_CT._

After exclusion of patients with LV systolic dysfunction Echo LV mass and CT LV mass were strongly correlated r = 0.85, *p* < 0.0001, however IVSd and CT LV mass were less so r = 0.50, *p* < 0.0001. Although body mass index >25 or >30 m/kg^2^ was not associated with troponin concentration, body mass index (BMI) ≥ 27.4 m/kg^2^ predicted the presence of LVH_Echo_ c-statistic 0.79, 95% confidence interval (CI) 0.68 to 0.89 (Figure 2a).

ROC analyses are presented in Table 2. Both the Singulex and Abbott troponin I assays were strongly diagnostic of LVH_Echo/CT,_ LAE and composite SHD (c = 0.74–0.87) and were superior to the Roche troponin I assay (c = 0.55–0.75), as well as NT-proBNP (c = 0.59–0.74) (Figure 2b,c). The optimal cut points were 1.5, 1.7, 6.5 ng/L and 6.3 pmol/L for predicting LVH_Echo_ for Singulex, Abbott, Roche assays and NT-proBNP respectively; 1.2, 1.4, 8.2 ng/L and 18 pmol/L for LAE_Echo_; and 1.2, 1.6, 8 ng/L and 18 pmol/L for composite SHD.

## 5. Discussion

In this study, we have shown that clinical factors such as age, gender and hypertension are strongly associated with troponin concentration, consistent with other data which has also shown an influence of physical activity, genetics and other factors including BMI on troponin [3]. Although in our study a BMI was not statistically associated with troponin concentration, a BMI ≥ 27.4 m/kg^2^ predicted the presence of LVH_Echo_. Our study has also found that hs-troponins, particularly Singulex and Abbott assays, as a stand-alone tool, to be strongly associated with echocardiographic markers of structural heart disease. As previously described NT-proBNP had a higher sensitivity and specificity for LV systolic dysfunction, compared to hs-troponin. From this one could infer that NT-proBNP would serve as clinically useful when used in conjunction with hs-troponin or in a sequential manner when screening populations [18]. After exclusion of patients with LV systolic dysfunction we found that hs-troponin was accurately identified patients with mild LVH diagnosed by echocardiography or CT. Our results are consistent with several other studies have also found hs-troponins to be associated with LVH [7,8,12,19,20,21,22]. In addition, we found male sex and hypertension were associated with higher hs-troponins, which was consistent with the findings of other studies [3,7]. As one would expect the presence of LAE was frequently found in patients with LVH and therefore by indirect association elevated hs-troponin was also associated with the presence of LAE. Similarly, one other study has shown a similar finding with hs-troponin being associated with LAE, as well as left ventricular diastolic diameter and diastolic dysfunction [8]. Since LV systolic dysfunction [12,19] and indirectly LAE are associated with an elevated troponin this may in part be the reason why high sensitivity troponin has a role in the prediction of atrial fibrillation [23] and stroke, in the context of proven atrial fibrillation [24,25]. 

In this small cohort, the number of patients with significant obstructive coronary disease was too limited to evaluate the relationship with hs-troponin. Whilst larger studies have shown a strong negative predictive value of Singulex troponin I (<1.53 ng/L) for the presence of obstructive coronary disease, causing ischemia, we could not demonstrate this finding due to the low numbers of patients in this study with obstructive coronary disease [11]. In addition, the concomitant presence of LVH in many patients with coronary disease, was an important confounder which will need to be considered when evaluating the diagnostic or prognostic utility of hs-troponin in stable exertional chest pain cohorts. We did however show that there was a strong correlation between Singulex and Abbott troponin I, thereby implying that outcome studies using one of these assays would also be applicable to the other. Concentration values and reference values however would need to be specific to each vendor. Whilst it is appreciated that the Singulex troponin has a higher sensitivity and precision that the Abbott troponin whether one assay has diagnostic superiority over the other would require a study of greater power than ours.

Our findings with those of others lend weight to the clinical applicability for ‘fourth generation’ hs-troponins. Apart from predicting cardiovascular events, failure and mortality, hs-troponins may be a useful screening tool for subclinical cardiac injury and dysfunction. This may provide a window of opportunity for earlier detection of LVH and act as a therapeutic guide. Two studies have shown that the addition of cardiac biomarkers, including hs-troponin with ECG and traditional cardiovascular risk factors, incrementally improves prediction of LVH [19,26]. Our cohort was too small to evaluate multivariate predictors of LVH. However, we found hs-troponins had high sensitivity and specificity to detect LVH_Echo/CT_/LAE_Echo_ as a stand-alone biomarker. In patients where white coat or mild hypertension is suspected hs-troponin could be used as a marker of end-organ damage, similar to the use of urine microalbumin creatinine ratio to detect hypertensive nephropathy.

Further research is required in large prospective studies to evaluate and determine the use of hs-troponins in cardiovascular screening. Multimarker studies are demonstrating that biomarkers such as troponin and BNP have utility in predicting coronary outcomes and stroke. Whether alterations in treatment, corresponding with a change in biomarker concentration then reflects a clinical positive clinical outcome remains uncertain, however one study did find decreased troponin concentrations correlating with reduced coronary events when treated with statins [15]. Longitudinal and interventional studies will be required to show whether response to treatment and reduction in hs-troponin correlates with regression of LVH. If high sensitive troponin is shown to quantitate LV mass and change in relation to treatment, it could then be used as a biomarker of treatment efficacy and a theranostic in the management of hypertension [14,27].

### Limitations

Our study has several limitations. The limited sample size of a pilot study meant not all significant associations could be found. It is an observational study which inevitably will contain bias. Note that patients included had low-intermediate risk chest pain so results may not be generalizable to asymptomatic healthy patients or with high-risk chest pain or established coronary artery disease and we did not have their final clinical diagnosis available to us at this point in time. Not all clinical characteristics were available for all patients, as these were collected as part of clinical care and their definitions were therefore open to clinician discretion. We also did not collect long-term outcomes to show the temporal adverse outcomes rates for this cohort.

## 6. Conclusions

In conclusion, hs-troponins, particularly Singulex and Abbott assays, were strongly predictive of LVH and LAE on echocardiography. Our study and others and advocate for the potential role for troponins as a screening tool for structural heart disease and larger studies are required to assess this to guide clinical decision-making.

## Figures and Tables

**Table 1:** Baseline characteristics and univariate associations with hs-troponin (Singulex assay) in total cohort. Mean ± standard deviation (SD), median (interquartile range) or number (%).

Characteristics	Mean ± SD
Age	58 (11)
	**Number (%)**
Male	29 (37)
Hypertension	40 (51)
Hypercholesterolemia	37 (47)
Diabetes	9 (15)
Smoking History	28 (36)
Family History of Cardiovascular Disease	26 (33)
Abdominal Obesity	27 (35)
Post-menopausal	32 (41)
**Imaging**	**Number (%)**
LVH_Echo_	29 (38%)
LAE_Echo_	29 (38%)
Composite SHD_Echo_	34 (45%)
LV Systolic Dysfunction	8 (11%)
Valvular Disease	8 (11%)
Coronary Artery Disease (CTCA)	32 (52%), 7 were obstructive
CT LV mass > 81.7 g/m^2^	15 (25%)
	**Median (IQR)**
IVSd	0.93 (0.79–1.1)
Echo LV mass	140 (105–179)
CT LV mass	135 (113–175)
Ejection fraction	58 (52–58)
Biomarkers	Median (IQR)
Singulex hs-Tn (ng/L)	1.2 (0.7–2.0)
Abbott hs-Tn (ng/L)	1.8 (1.1–3.3)
Roche hs-Tn (ng/L)	6.8 (3.7–10.3)
NT-proBNP (ng/L)	10 (4.7–22.9)

CT: computed tomography; CTCA: coronary artery disease; hs-Tn: high sensitivity troponin; IQR: interquartile range; IVSd: interventricular septal diameter; LAE: left atrial enlargement; LV: left ventricule; LVH: left ventricular hypertrophy; NT-proBNP: N-terminal pro-brain natriuretic peptide.

**Table 2:** Receiver-operative characteristics curve analysis of structural heart disease: figures are c-statistics (95% confidence interval (CI)).

	C-Statistic (95% CI)	Optimal Cut point (troponins ng/L, BNP pg/L)	Sensitivity	Specificity
**LV systolic dysfunction**				
NT pro-BNP	0.97 (0.9 to 0.99)	>29	100%	92%
Singulex troponin I	0.82 (0.71 to 0.91)	>1.7	83%	80%
Abbott troponin I	0.77 (0.66 to 0.86)	>12	50%	100%
**LVH_Echo_**				
Singulex TnI	0.84 (0.72–0.92)	>1.5	70%	88%
Abbott TnI	0.84 (0.73–0.92)	>1.7	85%	75%
Roche TnT	0.75 (0.63–0.85)	>6.5	77%	68%
NT-proBNP	0.62 (0.49–0.74)	>6.3	79%	53%
**LAE_Echo_**				
Singulex TnI	0.74 (0.6–0.85)	>1.2	71%	71%
Abbott TnI	0.78 (0.66–0.88)	>1.4	90%	53%
Roche TnT	0.55 (0.42–0.67)	>8.2	48%	69%
NT-proBNP	0.68 (0.62–0.85)	>18	52%	88%
**LVH_CT_**				
Singulex TnI	0.85 (0.72–0.93)	>2.1	62%	97%
Abbott TnI	0.87 (0.75–0.94)	>1.7	100%	59%
Roche TnT	0.75 (0.62–0.86)	>9	69%	80%
NT-proBNP	0.59 (0.45–0.72)	>6.3	75%	49%
**Composite SHD_Echo_**				
Singulex TnI	0.79 (0.66–0.88)	>1.2	74%	77%
Abbott TnI	0.82 (0.71–0.9)	>1.6	84%	68%
Roche TnT	0.62 (0.49–0.73)	>8	58%	70%
NT-proBNP	0.74 (0.62–0.84)	>18	58%	92%

## References

[1] Thygesen K., Alpert J.S., Jaffe A.S., Simoons M.L., Chaitman B.R., White H.D., Subcommittee B., Katus H.A., Apple F.S., Lindahl B. (2012). Third universal definition of myocardial infarction. Circulation.

[2] Blankenberg S., Salomaa V., Makarova N., Ojeda F., Wild P., Lackner K.J., Jørgensen T., Thorand B., Peters A., Petersmann M.N.A. (2016). Troponin I and cardiovascular risk prediction in the general population: The BiomarCaRE consortium. Eur. Heart J..

[3] Bossard M., Theriault S., Aeschbacher S., Schoen T., Kunz S., von Rotz M., Estis J., Todd J., Risch M., Mueller C. (2017). Factors independently associated with cardiac troponin I levels in young and healthy adults from the general population. Clin. Res. Cardiol..

[4] Wu A.H., Lu Q.A., Todd J., Moecks J., Wians F. (2009). Short- and long-term biological variation in cardiac troponin I measured with a high-sensitivity assay: Implications for clinical practice. Clin. Chem..

[5] Wu A.H., Akhigbe P., Wians F. (2012). Long-term biological variation in cardiac troponin I. Clin. Biochem..

[6] Petersen P.H., Fraser C.G., Sandberg S., Goldschmidt H. (1999). The index of individuality is often a misinterpreted quantity characteristic. Clin. Chem. Lab. Med..

[7] Aeschbacher S., Schoen T., Bossard M., van der Lely S., Glattli K., Todd J., Estis J., Risch M., Mueller C., Risch L. (2015). Relationship between high-sensitivity cardiac troponin I and blood pressure among young and healthy adults. Am. J. Hypertens..

[8] Ravassa S., Kuznetsova T., Varo N., Thijs L., Delles C., Dominiczak A., Diez J., Staessen J.A. (2015). Biomarkers of cardiomyocyte injury and stress identify left atrial and left ventricular remodelling and dysfunction: A population-based study. Int. J. Cardiol..

[9] Horwich T.B., Patel J., MacLellan W.R., Fonarow G.C. (2003). Cardiac troponin I is associated with impaired hemodynamics, progressive left ventricular dysfunction, and increased mortality rates in advanced heart failure. Circulation.

[10] Pascual-Figal D.A., Manzano-Fernandez S., Boronat M., Casas T., Garrido I.P., Bonaque J.C., Pastor-Perez F., Valdés M., Januzzi J.L. (2011). Soluble ST2, high-sensitivity troponin T- and N-terminal pro-B-type natriuretic peptide: Complementary role for risk stratification in acutely decompensated heart failure. Eur. J. Heart Fail..

[11] Tanglay Y., Twerenbold R., Lee G., Wagener M., Honegger U., Puelacher C., Reichlin T., Mann S., Druey S., Hochgruber T. (2015). Incremental value of a single high-sensitivity cardiac troponin I measurement to rule out myocardial ischemia. Am. J. Med..

[12] De Lemos J.A., Drazner M.H., Omland T., Ayers C.R., Khera A., Rohatgi A., Hashim I., Berry J.D., Das S.R., Morrow D.A. (2010). Association of troponin T detected with a highly sensitive assay and cardiac structure and mortality risk in the general population. JAMA.

[13] Saunders J.T., Nambi V., de Lemos J.A., Chambless L.E., Virani S.S., Boerwinkle E., Hoogeveen R.C., Liu X., Astor B.C., Mosley T.H. (2011). Cardiac troponin T measured by a highly sensitive assay predicts coronary heart disease, heart failure, and mortality in the Atherosclerosis Risk in Communities Study. Circulation.

[14] McEvoy J.W., Chen Y., Rawlings A., Hoogeveen R.C., Ballantyne C.M., Blumenthal R.S., Coresh J., Selvin E. (2016). Diastolic Blood Pressure, Subclinical Myocardial Damage, and Cardiac Events: Implications for Blood Pressure Control. J. Am. Coll. Cardiol..

[15] Ford I., Shah A.S., Zhang R., McAllister D.A., Strachan F.E., Caslake M., Newby D.E., Packard C.J., Mills N.L. (2016). High-Sensitivity Cardiac Troponin, Statin Therapy, and Risk of Coronary Heart Disease. J. Am. Coll. Cardiol..

[16] Everett B.M., Zeller T., Glynn R.J., Ridker P.M., Blankenberg S. (2015). High-sensitivity cardiac troponin I and B-type natriuretic Peptide as predictors of vascular events in primary prevention: Impact of statin therapy. Circulation.

[17] Klein R., Ametepe E.S., Yam Y., Dwivedi G., Chow B.J. (2017). Cardiac CT assessment of left ventricular mass in mid-diastasis and its prognostic value. Eur. Heart J. Cardiovasc. Imaging.

[18] De Lemos J.A., Ayers C.R., Levine B., deFilippi C.R., Wang T.J., Hundley W.G., Berry J.D., Seliger S.L., McGuire D.K., Ouyang P. (2017). Multimodality Strategy for Cardiovascular Risk Assessment: Performance in 2 Population-Based Cohorts. Circulation.

[19] Xanthakis V., Larson M.G., Wollert K.C., Aragam J., Cheng S., Ho J., Coglianese E., Levy D., Colucci W.S., Felker G.M. (2013). Association of novel biomarkers of cardiovascular stress with left ventricular hypertrophy and dysfunction: Implications for screening. J. Am. Heart Assoc..

[20] Koycheva R.Y., Cholakov V., Andreev J., Penev M., Iliev R., Nancheva K., Tsoneva V. (2016). Cardiac Biomarkers and Left Ventricular Hypertrophy in Asymptomatic Hemodialysis Patients. Open Access Maced. J. Med. Sci..

[21] Sun L., Tan X., Cao X., Zou J. (2016). Assessed value of high-sensitivity cardiac troponin T for cardiovascular disease among CKD patients. Ren. Fail..

[22] Ucar H., Gur M., Kivrak A., Koyunsever N.Y., Seker T., Akilli R.E., Türkoğlu C., Kaypakli O., Sahin D.Y., Elbasan Z. (2014). High-sensitivity cardiac troponin T levels in newly diagnosed hypertensive patients with different left ventricle geometry. Blood Press..

[23] Anegawa T., Kai H., Adachi H., Hirai Y., Enomoto M., Fukami A., Otsuka M., Kajimoto H., Yasuoka S., Iwamoto Y. (2012). High-sensitive troponin T is associated with atrial fibrillation in a general population. Int. J. Cardiol..

[24] Ruff C.T., Giugliano R.P., Braunwald E., Murphy S.A., Brown K., Jarolim P., Mercuri M., Antman E.M., Morrow D.A. (2016). Cardiovascular Biomarker Score and Clinical Outcomes in Patients With Atrial Fibrillation: A Subanalysis of the ENGAGE AF-TIMI 48 Randomized Clinical Trial. JAMA Cardiol..

[25] Hijazi Z., Oldgren J., Siegbahn A., Wallentin L. (2016). Application of Biomarkers for Risk Stratification in Patients with Atrial Fibrillation. Clin. Chem..

[26] Martinez-Rumayor A.A., de Lemos J.A., Rohatgi A.K., Ayers C.R., Powell-Wiley T.M., Lakoski S.G., Berry J.D., Khera A., Das S.R. (2013). Addition of highly sensitive troponin T and N-terminal pro-B-type natriuretic peptide to electrocardiography for detection of left ventricular hypertrophy: Results from the Dallas Heart Study. Hypertension.

[27] Bhatt D.L. (2016). Troponin and the J-Curve of Diastolic Blood Pressure: When Lower Is Not Better. J. Am. Coll. Cardiol..

